# Recent Developments in Two-Dimensional Materials-Based Membranes for Oil–Water Separation

**DOI:** 10.3390/membranes13070677

**Published:** 2023-07-18

**Authors:** Mohammadamin Ezazi, M. M. Quazi

**Affiliations:** 1 Department of Mechanical Engineering, Georgia Southern University, Statesboro, GA 30460, USA; 2 Faculty of Mechanical and Automotive Engineering Technology, Universiti Malaysia Pahang, Pekan 26600, Pahang, Malaysia; moinuddin@ump.edu.my

**Keywords:** membranes, oil–water separation, two-dimensional materials, MXene, graphene, metal–organic framework, covalent organic framework

## Abstract

The industrialization witnessed in the last century has resulted in an unprecedented increase in water pollution. In particular, the water pollution induced by oil contaminants from oil spill accidents, as well as discharges from pharmaceutical, oil/gas, and metal processing industries, have raised concerns due to their potential to pose irreversible threats to the ecosystems. Therefore, the effective treating of these large volumes of oily wastewater is an inevitable challenge to address. Separating oil–water mixtures by membranes has been an attractive technology due to the high oil removal efficiency and low energy consumption. However, conventional oil–water separation membranes may not meet the complex requirements for the sustainable treatment of wastewater due to their relatively shorter life cycle, lower chemical and thermal stability, and permeability/selectivity trade-off. Recent advancements in two-dimensional (2D) materials have provided opportunities to address these challenges. In this article, we provide a brief review of the most recent advancements in oil–water separation membranes modified with 2D materials, with a focus on MXenes, graphenes, metal–organic frameworks, and covalent organic frameworks. The review briefly covers the backgrounds, concepts, fabrication methods, and the most recent representative studies. Finally, the review concludes by describing the challenges and future research directions.

## 1. Introduction

The water pollution as a result of the rapid pace of industrialization in the last century has been steadily increasing and posing notable challenges worldwide [1,2]. The pollution of water by oils and organic contaminants, in particular, has been recognized as a major environmental concern since the pollution originates from multiple sources that can pose hazards not only to humans but also to terrestrial and marine ecosystems [3,4,5]. The oily wastewater can contain polyaromatic compounds and phenols that can impede the growth of species and cause cancer in humans [3]. These extensive effluents of oily wastewater are mainly originated from crude oil production and refineries, food production, textile processing, and oil spill accidents, which need to be treated in order to decelerate environmental degradation [6,7,8]. A variety of approaches have been implemented to treat oily wastewater including biological treatment, flotation, coagulation and flocculation, gravity separation, solvent extraction, and adsorption [9,10]. However, the majority of these methods may not provide an effective separation of emulsified oil–water mixtures, particularly when the size of oil droplets is lower than 20 µm [10,11]. Membrane-based technologies have been desirable for separating oil–water mixtures due to their size-exclusion mechanism that can provide selectivity for the effective separation of surfactant-stabilized emulsions [8,12]. Membrane-based separation also enables relatively low carbon footprints, scalability, continuous operation, and minimal requirements for chemical additives [13,14]. Another advantage of membrane-based technologies is their versatility and ability to undergo surface functionalization and wettability modifications, which makes them suitable for separating a broad range of wastewaters [6,15,16,17]. In particular, the surface wettability of the membranes can be modified by adjusting the solid surface energy and surface roughness to demonstrate superhydrophilic/superoleophobic or superhydrophobic/superoleophilic wettability for separating a wide range of emulsified oil–water mixtures [17,18,19,20,21]. Despite notable advancements in developing oil–water separation membranes, they need to be further improved in several aspects in order to meet the new complex requirements such as sustainable oily wastewater treatment [17,22,23]. These aspects include an improved fouling resistance and higher chemical and mechanical stability to realize membranes with longer life cycles in harsh environments [12,24]. In addition, the trade-off between selectivity and permeability has been another limitation of the membrane-based separation that requires immediate attention [24,25]. With tremendous recent developments in nanomaterials, particularly two-dimensional (2D) materials, unprecedented opportunities can be afforded to meet these new complex requirements in treating oily wastewaters [14,26,27]. Two-dimensional (2D) materials are ultrathin crystalline solids with a thickness of a few nanometers or lower that are characterized by significantly larger lateral dimensions in comparison to the thickness [28]. Such a large ratio of lateral dimensions to thickness enables the 2D materials to exhibit interesting properties in comparison to the bulk such as a high surface-to-volume ratio, tunable functionality, and a high strength that can exceed the strength of 3D materials [29,30]. These materials can be stacked to form a layered structure where the interlayer interactions are driven by weaker van der Waals forces [28,31]. The 2D materials with atomic-scale thickness can facilitate the permeate flux by minimizing the liquid transport resistance, which makes them desirable building blocks for preparing or modifying separation membranes [32]. The resulting membranes can also enable high selectivity as they consist of nano-scale channels with highly controllable pore structure and size [33]. In addition, these 2D materials-based membranes can provide a large specific surface area [26], and they have a high potential for modification of the surface wettability that makes them highly desirable for applications in oil–water separation.

In this brief review, we have provided a summary of the backgrounds, concepts, and fabrication methods, and we have discussed the most recent representative studies pertaining to the 2D materials-based membranes for oil–water separation. In particular, the contribution has focused on the most recent developments in the oil–water separation membranes that are modified with MXenes, graphene family materials, metal–organic frameworks (MOF), and covalent organic frameworks (COF) as common 2D materials.

## 2. MXene-Based Oil–Water Separation Membranes

### 2.1. MXene Materials and Their Use in Oil–Water Separation Membranes

MXenes represent a new category of two-dimensional (2D) transition metal carbides, nitrides, or carbonitrides characterized by a general formula of M_n+1_X_n_T_x_ [34,35]. In this formulation, M, X, and T represent early transition metals, nitrogen or carbon, and surface termination functional groups (e.g., hydroxyl, oxygen, fluorine), respectively [36]. Note that n is a number in the range of 1–4, while x denotes the number of functional groups [27,37]. More recently, MXenes have found potential applications in environmental remediation including the treatment of wastewater due to their attractive chemical, electronic, and mechanical properties [36]. These features include a large specific surface area, hydrophilic wettability, biocompatible properties, facile functionalization, tunable nanochannels, resistance to chemically and thermally aggressive environments, and facile regeneration [27,38,39,40,41]. The synthesis of MXenes has been conducted by employing two main approaches, bottom–up and top–down methods [42]. In the top–down approach, the large crystal quantities or the parent layered precursor is exfoliated to form single layers of MXene sheets [42,43]. This approach can be further divided into chemical and mechanical exfoliation/etching techniques. Common examples of chemical etching include electrochemical etching, hydrofluoric acid (HF) etching, and in situ HF etching [44]. Mechanical exfoliation has been reported as a less suitable method due to the strong metallic/covalent bonds of the parent MAX phases [45,46]. In contrast to the top–down method, the bottom–up approach is concentrated on growing MXenes from atoms or molecules [42,47,48]. The common methods of synthesizing MXenes via bottom–up approach include the template method [49,50], plasma-enhanced pulsed laser deposition [51,52], and chemical vapor deposition (CVD) [46]. In comparison to the methods that rely on etching, the bottom–up approaches, in particular CVD, can provide a final structure with high crystalline quality [46].

Membranes modified with MXenes have recently attracted particular attention as they can be utilized for separating oil–water mixtures with a high oil removal efficiency [9,27,53,54]. These membranes can be fabricated using different methods, including vacuum-assisted filtration and phase-inversion methods [27]. The surface roughness of the hydrophilic membranes modified with MXenes enables the retention of a water layer on the surface that can readily repel the oil phase. This can provide these membranes with an effective anti-fouling property that contributes to maintaining consistent oil–water separation functionality [55]. Furthermore, the nano-scale slit structure of these membranes along with their aligned morphology facilitates the permeation of the water phase that enables an effective separation from the retentate oil phase [27].

### 2.2. Recent Developments in MXene-Based Oil–Water Separation Membranes

**Figure 1:**
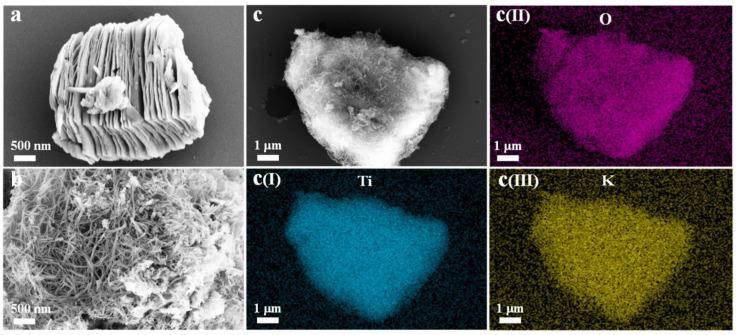
SEM images showing the structure of MXene and MNRs as well as the elemental mapping images. Note that (**a**) denotes MXene, while (**b**,**c**) represent MNRs. Reprinted from Ref. [56], Copyright (2022), with permission from Elsevier.

The abundance of hydrophilic functional groups contributes to promoting the performance of the MXene-based membranes [27]. This property can be further improved by incorporating design features that maximize the exposure of surface functional groups. A study by Huang et al., reported the modification of a mixed cellulose (MCE) membrane by utilizing fibrous MXene nanoribbons (MNRs) [56]. Figure 1a demonstrates the structure of the MXene, which resembles accordion-like morphology, whereas Figure 1b illustrates the fibrous morphology of the MNRs obtained after etching MXene by KOH [56]. The anti-adhesion capability and wettability of the membrane (MCE-MNR) were characterized by underwater oil contact angle (UOCA) measurements using diiodomethane. While MCE-MNRs demonstrated a maximum UOCA of 125°, the MCE-MXene showed a UOCA of 111°. The oil–water separation tests involved various types of oils: n-hexane, edible oil, gasoline, and diesel. The relatively larger pore size of MCE and MCE-MXene was found to be the main factor contributing to their lower separation performance [56]. In contrast, the nano-scale pores on the MCE-MNR membrane enabled a more effective separation with oil rejection rates of over 99% [56]. The improved performance of MCE-MNR was also attributed to the loose fibrous morphology of MNRs that enabled improved exposure of the surface functional groups and the formation of a water layer on the membrane surface that could effectively repel droplets of oil [56].

Overcoming the relatively low flux of the MXene-based membranes for oil–water separation is another arising area of interest. A study describes a fabrication approach that enables MXene lamellar membranes, which can separate oil-in-water emulsions at a high flux [57]. To enable a higher flux, expanded nanochannels were formed between the MXene nanosheets by intercalating the positively charged colloidal nanoparticles of Fe(OH)_3_ into the MXene nanosheets that bear negative charges [57]. Subsequently, hydrochloric acid treatment was carried out to remove colloidal nanoparticles and obtain a MXene membrane with higher interlayer spacing. This treatment process formed additional channels that facilitated the transport of fluids and enabled a higher flux. The results showed an increase in the pure water flux by a factor of two to nine compared to a pristine MXene membrane [57].

**Figure 2:**
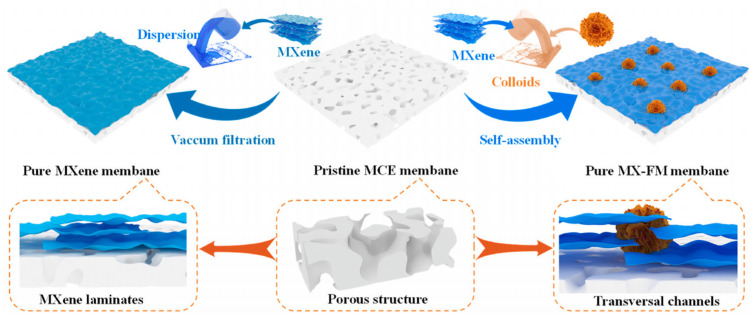
Schematic demonstration of the procedure employed to fabricate the MXa-FMb membrane. Reprinted from Ref. [58], Copyright (2023), with permission from Elsevier.

The relatively low flux of the traditional MXene-based membranes can be partly attributed to their dense slits and longer interlayer transport [58,59]. To address this challenge, a study investigated the integration of hydrothermally synthesized ferroferric oxide-doped molybdenum disulfide (FM) with a flower-like structure into MXene laminates via the self-assembly method [58]. As demonstrated in Figure 2, the FM dispersed in MXene solution was vacuum filtered onto the mixed cellulose (MCE) membrane, which was followed by drying at room temperature to obtain the final MXene-FM membrane (MXa-FMb) [58]. Consequently, the formation of transversal nanochannels, along with the surface roughness, enabled the membrane to demonstrate the permeance of ≈4.25 × 10^2^ L m^−2^ h^−1^ while separating the emulsions [58].

Similar to the conventional separation membranes, the contamination and fouling by organic contaminants and oil phases remains a major challenge that can compromise the separation performance of MXene-based oil–water separation membranes. Self-cleaning of the MXene-based oil–water separation membranes by incorporating photocatalysis technology has recently attracted escalating interest because it is an environmentally friendly method that can degrade organic contaminants into small molecules with minimal toxicity [54,57,60,61,62]. In a recent study, a composite MXene-based membrane was prepared for oil–water separation by incorporating MXene nanosheets and N-doped Bi_2_O_2_CO_3_ nanoparticles via a vacuum filtration process [54]. The results revealed that nanoparticles can enable a higher permeation flux by providing the MXene layers with additional channels for the permeation. The membrane demonstrated a water flux of 815.3 L m^−2^ h^−1^ and maintained rejection ratios of greater than 99% with oil–water emulsions prepared with different types of oils [54]. The photocatalytic capability of the membrane was evaluated by degrading different types of organic dyes in a solution phase under visible light irradiation. The dye removal reached 98.4%, 98%, and 99.9% for Rhodamine B, Trypan blue, and Congo red, respectively [54]. In addition, the self-cleaning ability of the membrane was investigated by performing the continuous filtration and photodegradation of Congo red solution. In the absence of light irradiation, the flux of dye solution through a photocatalytic membrane was reduced from 778.9 to 510.7 L m^−2^ h^−1^ after five cycles [54]. However, almost no decrease was observed in the flux of dye solution when the membrane was exposed to light irradiation. These results indicated in situ fouling removal via a photocatalysis mechanism [54].

Introducing the superwetting property is another alternative method to enhance the fouling resistance of the membranes. To capitalize on this property, a study employed Ca^2+^-crosslinking to fabricate Ti_3_C_2_T_x_ MXene membranes intercalated with hydrophilic sodium alginate [63]. The presence of sodium alginate enabled larger MXene nanochannels and introduced superwetting properties with underwater oil contact angles measured at 151° as an indication of very low oil–membrane adhesion [63]. While the pristine MXene membrane showed a significant flux decrease from 5.77 to 0.54 L m^−2^ h^−1^ bar^−1^ after five cycles of stabilized oil–water emulsion separation, the flux of the membrane modified with sodium alginate remained nearly constant, indicating a superior anti-fouling characteristic [63].

Table 1 summarizes the main findings for the most recent MXene-based oil–water separation membranes.

## 3. Graphene-Based Oil–Water Separation Membranes

### 3.1. Graphene Materials and Their Use in Oil–Water Separation Membranes

Graphene is a single free-standing layer of sp2-hybridized carbon atoms that are bound in the hexagonal honeycomb array [64,65,66,67]. Other materials that are related to graphene include graphene oxide (GO), reduced graphene oxide (rGO), and ultrathin graphite [68,69]. Although graphene and GO have similar structures, the use of strong acids and oxidants in the preparation of GO introduces several oxygen-containing functional groups such as carboxyl, hydroxyl, and carbonyl to the GO structure [70]. The GO can be transformed into rGO via a reduction process that decreases the oxygen content and results in a structure with enhanced thermal and chemical stability [70,71]. As a result of its distinctive geometry and structure, graphene-based materials can demonstrate exceptional physico-chemical properties including a high Young’s modulus, thermal and electrical conductivity, high fracture strength, chemical inertness, and tunable functionality [71,72,73,74,75,76]. In particular, graphene-based materials can offer extensive utility in environmental remediation applications, including the removal of waterborne pathogens, heavy metals, and organic contaminants due to their extremely high surface area that can even exceed the surface area of well-developed activated carbons [77,78]. Various methods have been developed to synthesize graphenes, which include micromechanical cleavage or the scotch tape method [79], liquid-phase mechanical exfoliation [80], chemical cleavage and exfoliation [81], epitaxial growth [82], and chemical vapor deposition (CVD) [66,81,83,84]. Among these synthesis approaches, the micromechanical cleavage remains a popular approach due to its simplicity that can be implemented without specialized equipment [81]. Despite simplicity, this method is inefficient in mass producing graphenes for various applications [81]. Hence, liquid-phase sonication methods have been implemented to facilitate the generation of ultrathin carbon films [81]. Furthermore, the advantage of the CVD approach is the ability to produce polycrystalline single-layer graphene over a large area with an exceptional quality [81,83,84]. Graphene-based materials have been extensively utilized to modify porous substrates in order to obtain the desired liquid wettability. Among the substrate materials, the use of polyvinylidene fluoride (PVDF) and cellulose acetate (CA) has been dominant due to their mechanical strength, oxidation resistance, and thermal stability [85,86]. In particular, the modification of PVDF substrates has been the center of investigation, as their hydrophobic wettability makes them prone to fouling and shortens their life cycle [86,87,88]. The membranes modified by graphene-based materials can be prepared mainly by two methods, vacuum filtration and electrospinning [85]. While in the vacuum filtration approach, the suspension of graphene-based nanomaterials is vacuum-filtered on a given substrate membrane, the electrospinning utilizes the precursor liquid to weave electrospun fibers into a membrane substrate [85].

The exceptional mechanical properties of graphene-based materials along with their high surface area make them suitable for fabricating membranes or modifying porous structures for applications in oil–water separation. These materials can form micron and nano-scale roughness on a surface to enable enhanced wetting properties. A main strategy in utilizing graphene-based materials is to modify meshes, aerogels, or sponges to introduce superhydrophobic–superoleophilic wettability, or to fabricate materials that enable superhydrophilic–underwater superoleophobic wettability [85].

### 3.2. Recent Developments in Graphene-Based Oil–Water Separation Membranes

**Figure 3:**
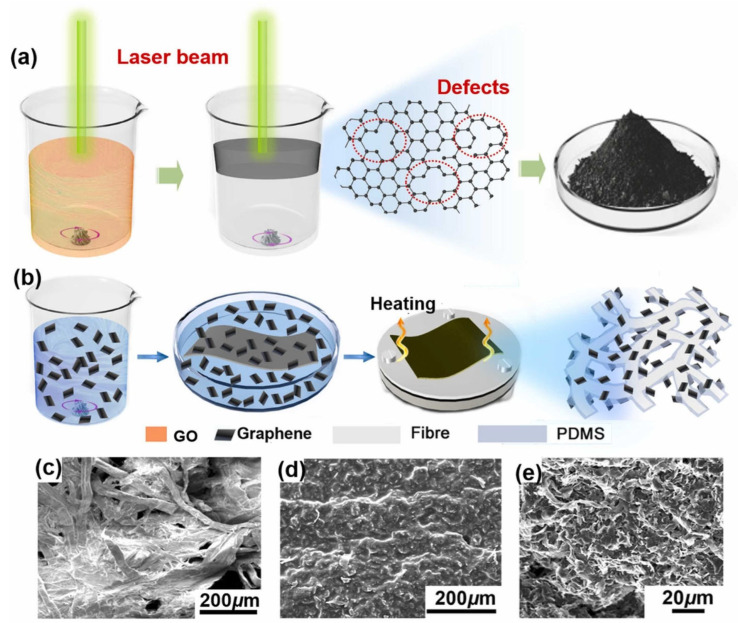
Schematic illustrations of (**a**) laser-aided reduction of GO, and (**b**) fabrication of composite paper. SEM images of (**c**) an uncoated paper tissue and (**d**) composite paper. (**e**) Magnified view showing the surface of composite paper. Reprinted from Ref. [89], Copyright (2022), with permission from Elsevier.

In pursuit of performing oil–water separation at high flux and efficiency, a study by Sun et al., reported the fabrication of a composite paper by coating laser-aided reduced graphene oxide and hydrophobic polydimethylsiloxane (PDMS) onto an inexpensive paper tissue, as shown in Figure 3a,b [89]. Figure 3c–e demonstrate SEM images of uncoated paper tissue and the one after coating, respectively [89]. The laser was utilized to obtain hydrophobic graphene by reducing the graphene oxide without needing to introduce hazardous reducing chemicals. The laser irradiation also enabled the formation of defects on graphene that contributed to a 25% increase in the specific surface area [89]. The strong capillary force provided by the paper tissue along with the superhydrophobic wettability of the functional coating enabled the separation of surfactant-stabilized water-in-oil emulsions with an efficiency of up to 99.85% and flux of 4421 L m^−2^ h^−1^ [89].

In another study inspired by cell membranes, nanomesh membranes were developed with improved permeance and anti-fouling property by employing the vacuum-aided self-assembly method [90]. The nanopores were etched on graphene oxide (GO) by utilizing a partial combustion method, which reduced the length of the water transfer channels. The presence of graphene with hydrophobic wettability along with the modification using hydrophilic chitosan enabled the membrane to demonstrate low friction with the water permeate and a low oil adhesion underwater [90]. The enhanced water transport along with anti-fouling property enabled the membrane to show a water permeance of up to ≈4000 L m^−2^ h^−1^ bar^−1^ [90].

In regard to enhanced fouling resistance, Yang et al., developed a GO-based membrane that can demonstrate outstanding anti-fouling properties even at high permeance [91]. The membrane was fabricated by utilizing a GO membrane on which the layers of hydrophilic phytic acid (PA) and hydrophobic perfluorocarboxylic acids were consecutively assembled. To reduce the surface energy while tuning the surface hydration, perfluorocarboxylic acids were utilized with varied lengths of hydrophobic chains [91]. This approach resulted in a surface with an ability to demonstrate both the resistance against fouling as well as fouling release properties, which is conventionally challenging [92]. The membrane demonstrated outstanding fouling resistance as characterized by a flux decline ratio of lower than 10% at a permeance of ≈620 L m^−2^ h^−1^ bar^−1^ [91].

Maintaining the structure stability of the lamellar GO layer can be challenging due to its tendency to disintegrate in an aqueous environment [93]. In view of this challenge, a multifunctional fibrous composite membrane was fabricated by spray coating three-dimensional (3D) TiO_2_@crumpled graphene oxide core–shell spheres on a porous support made of electrospun poly(acrylene ether nitrile) (PEN) [93]. Note that polydopamine was also utilized to mediate crosslinking and enhance the durability of the functional layer [93]. The physical stability and well-regulated nanochannels of the membranes enabled a high permeance of 3142–3514 L m^−2^ h^−1^ for oil-in-water emulsions and a rejection rate in excess of 99% [93].

Integrating switchable wettability into separation membranes has been another intriguing line of research that can enable an on-demand separation of oil and water [94,95,96]. However, fabricating graphene-based membranes that can demonstrate on-demand oil–water separation has been challenging. To address this challenge, a study investigated the in situ deposition of surfactant-modified graphene oxide (GO-CTAB) on the surface of different metal meshes by employing an electrodeposition technique [94]. The membrane demonstrated a reversible wettability switch from hydrophilic and underwater superoleophobic to superhydrophobic when exposed to organic solvents due to conformational changes in surface molecules. The membrane could retrieve the hydrophilic wettability upon applying a negative electrical potential [94]. The switchable wettability of the membrane enabled it to separate both water-in-oil and oil-in-water emulsions under gravity [94].

Table 2 summarizes the main findings for the most recent graphene-based oil–water separation membranes.

## 4. Metal–Organic Framework (MOF)-Based Oil–Water Separation Membranes

### 4.1. Metal–Organic Framework (MOF) Materials and Their Use in Oil–Water Separation Membranes

Metal–organic frameworks (MOFs) are an emerging category of highly crystalline and porous materials that are formed by assembling organic linkers and metal nodes [97,98,99,100]. The organic linkers can be ditopic or polytopic polyamines, phosphonates, cyano groups and carboxylates, which can form robust 2D or 3D crystalline structures when linked by the metal nodes [101,102]. The MOFs can provide highly ordered structures that enable them to demonstrate unique properties such as the high surface area, which can even exceed 6000 m^2^/g, thermal and chemical stability, low density, and versatility of the structure, all of which makes them suitable for a broad range of applications [97,99]. The structure of MOFs can be readily modified during the synthesis process by controlling the synthesis technique, synthesis conditions such as the pH and temperature, and the starting materials including the organic linkers and metal precursors [103,104]. MOFs are recognized for providing high porosity with small uniform pore size, which can be readily functionalized to benefit mass transport and separation applications, particularly membrane-based separation [103,105]. Fabricating or modifying porous membranes by MOFs has been realized using different synthesis approaches including the hydrothermal method [106], electro-spinning [107,108], layer-by-layer (LBL) coating [109,110], phase inversion [111], vacuum-aided filtration [112,113], and solvothermal method [97,114,115]. Among these approaches, the hydrothermal method can provide a more straightforward and environmentally benign synthesis route with a higher cost efficiency [106]. The LBL approach capitalizes on the sequential submerging of substrate material in solutions that contain metal ions or organic ligands [97]. The preparation of the membrane using the phase inversion method involves a well-stirred homogeneous solution that contains both organic ligands and metal ions. The solution is cast followed by pulling into a water bath to enable immersion precipitation [111]. Finally, in the case of the solvothermal method, the substrate material is submerged in solutions of organic ligands and metal ions followed by treating the substrate at an elevated temperature in an autoclave [116].

Conventionally, polymeric separation membranes have dominated the market as they can be prepared at a relatively low cost. Also, these membranes are capable of demonstrating promising permeate fluxes and mechanical flexibilities [105,117]. However, the relatively short life cycle of these membranes, low thermal and chemical stability, and low selectivity have limited the practicality of these membranes for oil–water separation [105]. In contrast, the membranes fabricated or modified by MOFs have been very appealing for oil–water separation applications due to their uniform pore structure that can be readily controlled by the interplay of organic ligands and metal ions, chemical and thermal stability, as well as their ease of functionalization [105,118].

### 4.2. Recent Developments in MOF-Based Oil–Water Separation Membranes

**Figure 4:**
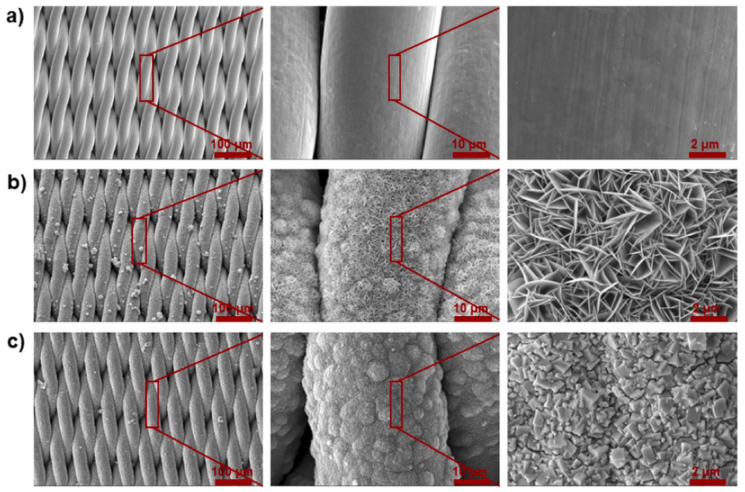
SEM images of (**a**) stainless steel metal mesh, (**b**) Ni/Al layered double hydroxides (LDH) grown on metal mesh, and (**c**) uniform layer of MOF-303 formed on metal mesh. Reprinted from Ref. [119], Copyright (2023), with permission from Elsevier.

Simultaneous fouling by insoluble oil droplets and organic dyes in wastewater has been an area of continuous investigation to develop more effective membranes that can maintain consistent flux. In a study by Yin et al., a stainless-steel metal mesh was utilized as a porous substrate to be coated with MOF-303 [119]. A facile and economical hydrothermal method was utilized to coat the well-ordered structure of MOF-303 crystals. Figure 4a shows the surface of the metal mesh before the coating process [119]. Figure 4b demonstrates the surface of the metal mesh after the in situ growing Ni/Al layered double hydroxides (LDH), which served as the precursor to facilitate the nucleation of MOF-303 [119]. Subsequently, a film of MOF-303 was formed that uniformly covered the metal mesh surface, as shown in Figure 4c [119]. The unique structure of MOF-303 combined with the abundant presence of hydrophilic carboxylate functional groups enabled an excellent water affinity [119]. Consequently, the membrane could form a hydration layer to demonstrate an effective anti-fouling property. The membrane demonstrated the gravity-driven separation of the oil–water mixture with a flux of >12308 L m^−2^ h^−1^ and a separation efficiency greater than 99.35%. Furthermore, the membrane could effectively remove water-soluble dyes with >99% efficiency [119].

Integrating anti-fouling properties into oil–water separation membranes via photo-Fenton catalytic mechanism is another approach to maintain a consistent separation permeate flux and efficiency [120,121]. A study employed a quartz fibrous membrane with a high structural and thermal stability as the substrate to grow NH_2_-MIL-88B MOF via a one-step solvothermal method [114]. The prepared MOF provided the hierarchical structure necessary to achieve superhydrophilic and underwater superoleophobic wettability. Furthermore, the iron-based MOFs can provide excessive numbers of hydrophilic amino functional groups and can generate reactive •OH radicals to demonstrate self-cleaning capability [114,122,123]. The membrane could demonstrate a separation efficiency and flux of up to 99.4% and >350 L m^−2^ h^−1^, respectively, for a range of surfactant-stabilized oil–water mixtures [114]. The reduced flux of the membrane due to the formation of oil “filter cake” could be recovered to some extent via washing by water. Ultimately, the photo-Fenton-driven self-cleaning of the membrane under visible light enabled the near-complete recovery of the water flux [114].

In addition to anti-fouling properties, the quest for membranes that can provide ultrafast oil–water separation has been on the rise to meet the demand in applications that produce large volumes of oil–water mixtures such as mining operations [124]. Recently, an ultrafast oil–water separation membrane was developed by growing 2D Cu triphenylene catecholate MOF on a copper mesh [125]. The special hierarchical structure, along with the polar nano-cavity in the MOF crystal, enabled effective superhydrophilic and underwater superoleophobic wettability [125]. Consequently, the membrane demonstrated the gravity-driven separation of different oils including actual crude oil. The membrane enabled an ultrahigh flux of up to 329 kL m^−2^ h^−1^ during the separation process [125]. In addition, the unique hierarchical structure of the membrane contributed to an improved separation efficiency with permeates showing less than 24.6 mg L^−1^ of oil contents [125].

Despite the advancements in developing high-flux MOF-based membranes for oil–water separation, a majority of them can demonstrate only a single type of surface wettability, which may not meet the complex requirements in the sustainable treatment of wastewater [17]. This challenge can be partly addressed by developing membranes that can separate oil–water mixtures on demand. Incorporating switchable wettability has been a promising approach to realize on-demand separation capabilities. In such membranes, an alternation between hydrophobic and oleophobic wettability can be acquired under the influence of external triggers such as pH change [126,127], light irradiation [128,129], temperature change [130], and electricity [17,131]. In a study, a separation membrane with switchable superwettability was developed by growing CAU-10-H MOF on stainless steel mesh surface via employing the solvothermal method [17]. The interpenetrating hierarchical structure of the CAU-10-H crystals enabled dual superlyophobicity in submerged conditions. Prewetting the membrane with water resulted in the formation of a trapped layer of water that could prevent oil permeation, whereas the initial wetting by oil enabled a trapped oil layer that resisted water permeation [17]. This property enabled the membrane to demonstrate the on-demand separation of different stabilized oil–water emulsions with a flux of more than 1.85 × 10^5^ L m^−2^ h^−1^ and >99.9% of separation efficiency [17].

Another line of research with rising popularity is the development of sustainable MOF-based membranes by utilizing biodegradable and renewable materials. These metal–organic frameworks are referred to as Bio-MOFs, which are synthesized by utilizing saccharides, peptides, and amino acids [132]. A study reported the preparation of a superhydrophobic Bio-MOF coating on the surface of fabric for oil–water separation [133]. The Bio-MOF was prepared by employing an electrochemical process and utilizing aspartic acid as the ligand and copper cores to obtain a rough surface, which was followed by treatment with stearic acid to lower the solid surface energy [133]. Subsequently, the Bio-MOF was sprayed onto the fabric surface to obtain the separation membrane. The membrane could demonstrate separation efficiency in the range of 95–99.4% and flux rates between 15,400 and 15,700 L m^−2^ h^−1^ for different oil–water mixtures [133].

Table 3 summarizes the main findings for the most recent MOF-based oil–water separation membranes.

## 5. Covalent Organic Framework (COF)-Based Oil–Water Separation Membranes

### 5.1. Covalent Organic Framework (COF) Materials and Their Use in Oil–Water Separation Membranes

Covalent organic frameworks are a new class of crystalline porous materials that are formed by combining completely organic building blocks which are linked via covalent bonds to establish a highly ordered extended structure of 2D or 3D crystalline solids [134,135,136]. These organic building blocks are typically composed of light elements such as Si, C, N, O, and B [134]. While the 3D COFs are linked entirely by covalent bonds throughout the three-dimensional structure, the covalent bonds are present exclusively within the conjugated two-dimensional sheets in 2D COFs, and the layers are held by weak interactions such as hydrogen bonds and π–π stacking [137]. A covalent organic framework can offer multiple benefits including large surface area, chemical stability, low density, and ease of functionalization [137]. The reticular chemistry of COFs and their tunable functionality have diversified their applications across various fields ranging from separation to energy sectors [138]. Perhaps separation is among the applications that have most benefited from the advancements in COFs, as they have the potential to offer promising solutions to the permeability/selectivity trade-off due to their high porosity, well-defined and tunable pore size, and adjustable surface functionality [139]. Among separation methods, membrane-based separation can be significantly advanced by COFs, as the current polymer-based membranes typically suffer from the trade-off between permeability and selectivity [139]. In fact, the crystalline structure of the COFs with the orderly arranged pores act as transport channels for the liquids to permeate efficiently. Nevertheless, fabricating membranes that can take full advantage of such an orderly structure has been a challenge [139]. In particular, the in situ growth of defect-free COF layers on porous substrates has been challenging due to their limited film-forming ability [139]. Fabricating free-standing COF membranes has been a strategy to address the inadequate film-forming ability of COFs on substrates [140,141]. Consequently, a mechanically robust free-standing COF membrane requires thickness in the order of hundreds of micrometers, which can significantly compromise the permeate flux [139]. In view of these challenges, several other fabrication approaches have been implemented. For example, COFs can be integrated into common membrane fabrication methods such as non-solvent-induced phase inversion (NIPS) [142]. The presence of COFs provides additional liquid passage channels to improve the permeability, which is traditionally provided by the pores that are formed during the solvent exchange process [143]. Stacking the nanosheets of COF is another approach to fabricate COF-based membranes [143]. In this method, the bulk material is initially exfoliated in order to generate nanosheet dispersions in a solvent. Subsequently, dip coating or vacuum filtration methods are employed to form the COF layer on top of a porous substrate [143]. Interfacial polymerization (IP) is another method of fabricating COF-based membranes that utilizes a relatively mild reaction condition [144]. This method has evolved as a facile approach wherein the interface of two immiscible phases containing the monomers is utilized to perform the polymerization reaction [145,146]. It is worth noting that the final membrane structure is a function of the reactivity of the monomers [146]. The advantages of the interfacial polymerization method include the ability to fabricate thin membranes with relatively high permeability as well as the high scalability that makes it highly compatible with automatic membrane manufacturing [143,144].

Despite the use of COF-based membranes in liquid phase separation such as seawater desalination [147,148], removal of toxic ions [149,150], and organic contaminants removal from water [151,152], only a limited number of studies have reported the application of COF-based membranes in oil–water separation.

### 5.2. Recent Developments in COF-Based Oil–Water Separation Membranes

Incorporating selective wettability has been a promising approach to obtain a membrane for oil–water separation applications [153,154]. In a study, a dip-coating method was implemented in order to form Schiff base COFs (Tp-BD and TAPB-TPA) on electrospun nanofibers of polyacrylonitrile (PAN) [155]. The rough nanoscale structure along with hydrophobic modification using alkyl (Lauryl) groups resulted in a surface with superhydrophobic and oleophilic wettability. The water contact angle was measured as ≈167° on the surface of the membrane [155]. The membrane could separate the oil–water mixtures that were prepared using different oil types and concentrations with efficiencies greater than 95% [155].

**Figure 5:**
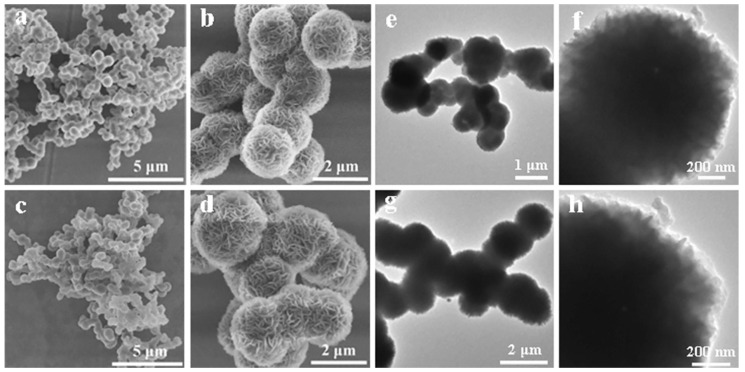
SEM images of (**a**,**b**) imine-linked COF (COF-LZU1), and (**c**,**d**) quinoline-linked COF with -CF_3_ groups (COF-Q-CF_3_). TEM images of (**e**,**f**) imine-linked COF (COF-LZU1), and (**g**,**h**) quinoline-linked COF with -CF_3_ groups (COF-Q-CF_3_). Reprinted from Ref. [156], Copyright (2022), with permission from Elsevier.

Preserving the stability of the COFs following the hydrophobic modification can be challenging, as it may affect the porosity, crystallinity, and morphology of the COFs. In a study by Wang et al., 2D imine-linked COFs with hydrophilic wettability were converted into aromatic quinoline-linked COFs with hydrophobic wettability by reacting enamides and imines through an aza-Diels–Alder process [156]. Figure 5a,b and Figure 5e,f show the SEM and TEM images of the imine-linked COF (COF-LZU1), respectively [156]. Figure 5c,d and Figure 5g,h, respectively, exhibit the SEM and TEM images of the quinoline-linked COF with -CF_3_ groups (COF-Q-CF_3_) [156]. The porosity and crystallinity of the COFs were maintained despite a significant increase in the hydrophobic wettability after the conversion. In particular, the quinoline-linked COF with -CF_3_ groups demonstrated superhydrophobic wettability, which is attributed to the low solid surface energy of the perfluorinated groups [157].

**Figure 6:**
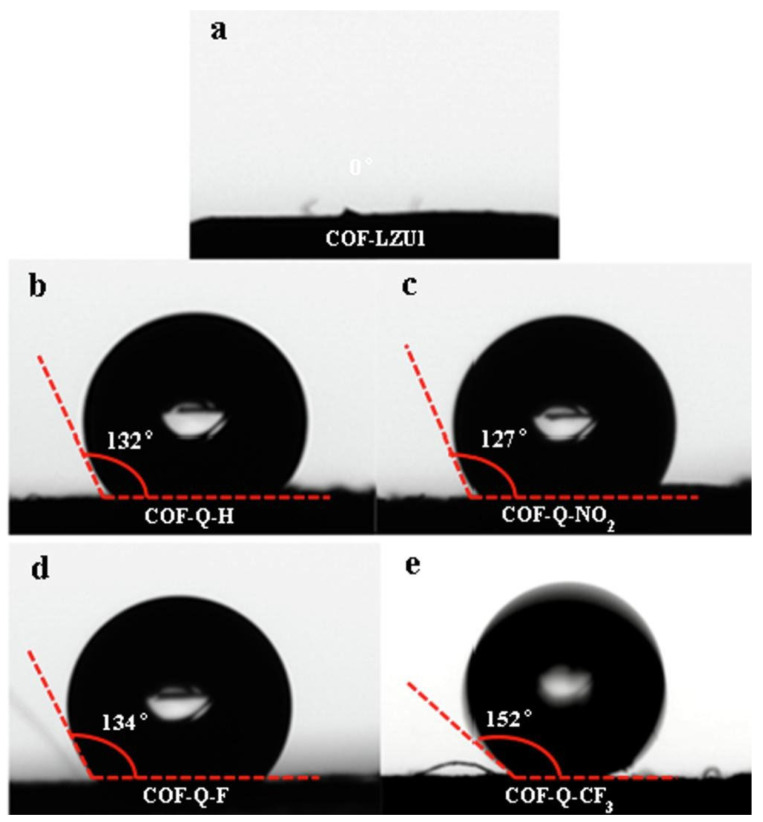
Contact angles of water on the pressed pellets of (**a**) imine-linked COF (COF-LZU1) and (**b**–**e**) quinoline-linked COFs. Reprinted from Ref. [156], Copyright (2022), with permission from Elsevier.

Figure 6 shows the contact angles of water on the surface of COF-LZU1 and the quinoline-linked COF pellets [156]. The oil–water separation capability of the COF layer was tested using water-in-oil emulsions. The results showed that the oil phase in the emulsion could readily permeate the dense layer of COF, whereas the water phase was retained above the COF layer [156]. The separation efficiency reached about 99.6% for water-in-chloroform emulsion, and the oil permeation flux for different emulsions ranged from 1.1 × 10^4^ to 2.2 × 10^4^ L m^−2^ h^−1^ when using the quinoline-linked COF with -CF_3_ groups [156].

In another study, oil–water separation membranes were prepared by growing 2D COFs on stainless steel net substrates via condensation reaction of fluorine and/or isopropyl functional groups and perfluorodialdehyde with triamines [158]. The membrane separated oil–water mixtures by allowing the permeation of oil while repelling water. Results showed that the separation efficiency and permeation flux of the membrane can reach over 99.5%, and 2.84 × 10^5^ L m^−2^ h^−1^, respectively [158].

The functionalization of imine-linked COFs with fluorine atoms to enhance hydrophobic wettability may be challenging due to their limited reactivity, which can make the large-scale manufacturing of the final product economically less feasible [159]. To address this challenge, a study reported a rapid microwave-aided synthesis method to form imine-linked COFs via the condensation of 1,3,5-tris(4-aminophenyl)benzene (TAB) with pyridine-based aldehyde linkers. The process imparted thioanisole functional groups into the building blocks of the COF, which resulted in hydrophobic wettability [159]. This method is synthetically more straightforward compared with introducing fluorine atoms via postsynthetic material modification approaches [159]. The resulting COF demonstrated superhydrophobic wettability to enable oil adsorption and water repellency. The adsorbed oil could be readily removed via rinsing by oleophilic solvents such as diethyl ether to regenerate the COF [159].

In another work, the carboxylated COF (COF-COOH) integrated with polydopamine (PDA) was assembled on the surface of the PVDF microfiltration membrane by employing a dip-coating method [160]. The high interfacial compatibility between COF-COOH and PDA resulted in a uniform and stable coating with high bond strength. The hierarchical structure along with the hydrophilic carboxyl groups enabled the membrane to demonstrate superhydrophilic and underwater superoleophobic wettability [160]. The membrane showed an oil rejection ratio of >98% and the permeate flux of up to 1843.48 L m^−2^ h^−1^ bar^−1^ [160].

Table 4 summarizes the main findings for the most recent COF-based oil–water separation membranes.

## 6. Future Directions

The separation of liquids by membranes has attracted considerable interest due to the relatively low energy requirement, continuous operation, minimum requirements for chemical additives, and size exclusion-driven selectivity. In particular, membrane-based technologies have found unparalleled application in treating oily wastewater, as their surfaces can be functionalized and undergo roughness modifications to enable the separation of various types of oil–water mixtures at different permeate fluxes. Conventionally, polymers have been utilized as the main material to develop these membranes as they can be processed using relatively straightforward fabrication techniques at reasonably low prices. Moreover, the use of polymers in these membranes enables a mechanically flexible structure that can meet the ordinary requirements in separating oil–water mixtures. However, challenges such as the relatively low chemical and thermal stability as well as the permeability-selectivity trade-off have prompted extensive research efforts to find replacements. Furthermore, with the ever-increasing need to develop more efficient solutions to treating complex industrial oily wastewater, the development of next-generation oil–water separation membranes has become inevitable. With the recent advancements in the synthesis and applications of two-dimensional (2D) materials, unprecedented opportunities have emerged to meet these new complex requirements. Two-dimensional (2D) materials can offer unique characteristics including high surface area, relatively uniform pore size, high porosity, and tunable wettability. Owing to these characteristics, 2D materials have been utilized to develop membranes, which can demonstrate promising oil–water separation efficiency and flux. Despite significant advancements, there are several key aspects of 2D materials-based membranes that require further research, as summarized below.

### 6.1. Membrane Thickness

Membrane thickness is a critical factor that affects the separation performance of a membrane [18]. A lower thickness of 2D materials-based membranes would not only contribute to a higher permeate flux and mass transport but will also reduce energy consumption. However, reducing the thickness of a membrane can compromise mechanical durability, as the membrane may require to be prepared free-standing and without using the porous supporting substrate. In particular, the mechanical durability becomes nontrivial when the membrane is subjected to high shear forces by the feed stream in cross-flow configurations [120]. Hence, further research is required to realize ultrathin free-standing 2D materials-based oil–water separation membranes with high mechanical durability.

### 6.2. Membrane Cytotoxicity and Long-Term Stability

Future research should also further study the cytotoxicity of the 2D materials-based membranes through numerous cycles of separation to investigate their practicality for actual oily wastewater treatment [27,161]. The long-term stability is also of interest for future investigations. Studies are encouraged to further investigate the long-term performance of these membranes particularly in aggressive environments with extreme pH levels, high temperatures, and high salinity.

### 6.3. Multifunctional Membranes

Incorporating additional functionalities into the 2D materials-based oil–water separation membranes is another line of research that can be further investigated to meet the more stringent future demands in industrial wastewater treatments. These additional functionalities include visible light-driven photocatalytic self-cleaning, removal of other contaminants (e.g., heavy metals, pesticides, pharmaceuticals), selectivity, and stimuli-responsive wettability.

### 6.4. Large-Scale Fabrication

Finally, research efforts are encouraged to investigate the possibility of facilitating the larger-scale fabrication of these membranes at a higher rate while lowering the nanopores defects. With the recent advancements in synthesis and manufacturing technologies including advanced robotics, precision 3D printing, and other non-conventional methods, significant breakthroughs are expected in the large-scale production of these membranes at lower prices [14,162].

## 7. Conclusions

This brief review aimed at summarizing the most recent advancements in developing membranes modified with two-dimensional (2D) materials for applications in oil–water separation. In particular, the contribution was focused on MXenes, graphene family materials, metal–organic frameworks (MOF), and covalent organic frameworks (COF) as the most common synthetic 2D materials that have been utilized to fabricate or modify the separation membranes. The characteristics of each 2D material along with the common synthesis/fabrication methods and the most recent representative studies were discussed. The 2D materials can provide unique characteristics including large specific surface area, high porosity, uniform pore size, tunable wettability, facile functionalization, tunable nanochannels, resistance to chemically and thermally aggressive environments, and mechanical durability. These unique characteristics have enabled the development of promising membranes that can demonstrate high permeate flux, improved separation efficiency, and promising regeneration capabilities when separating both free and emulsified oil–water mixtures. Despite these advancements, several limitations are identified for these membranes such as mass production, large-scale fabrication, and nanopores defects. With the anticipated tremendous progress in manufacturing technologies, materials synthesis, and computational methods, it is expected that most of these restrictions will be addressed in the near future.

## Figures and Tables

**Table 1:** Summary of the main findings for the most recent developments in MXene-based oil–water separation membranes.

Membrane Materials	Synthesis/Fabrication Methods	Wettability	Permeate Flux	Type of Oils Separated	Separation Efficiency/Rejection Rate	Characteristics, Strengths, and Shortcomings
(i) MXene						
[56] Fibrous MXene nanoribbons (MNRs) and mixed cellulose (MCE)	Vacuum filtration	Superhydrophilic + Underwater oleophobic (UOCA of 125°)	15,860.24 L m^−2^ h^−1^ bar^−1^	Oil–water mixtures n-hexane, edible oil, gasoline, diesel	>99%	By using ultraviolet light irradiation, photocatalytic self-cleaning can effectively increase the flux recovery rate (FRR).
[57] MXene nanosheets and porous polyvinylidene fluoride (PVDF) substrate and photocatalyst β-FeOOH	Vacuum filtration and in-site mineralization Intercalating the positively charged colloidal nanoparticles of Fe(OH)_3_	Superhydrophilic (WCA = 0°) + Underwater superoleophobic (UOCA ~ 151–160°)	~500.3–1022.7 L m^−2^ h^−1^	Oil-in-water emulsions Crude oil, isooctane, n-hexane, toluene, petroleum ether, 1,3,5-trimethyl benzene, dichloromethane	>99%	Self-cleaning capabilities due to photo-Fenton property, and low oil adhesion. Chemically stable under conditions of high temperature (90 °C), high salinity, and severe corrosion.
[58] MXene laminates embedded with ferro-ferric oxide doped molybdenum disulfide flower-like composites	Facile fabrication strategy with vacuum filtration and self-assembly	Hydrophilic (WCA ~ 64.4–74.5°)	Oil mixtures 3.75 × 10^3^ L m^−2^ h^−1^ Emulsions 4.25 × 10^2^ L m^−2^ h^−1^	Oil–water mixtures and emulsions n-hexane and edible oil	>99%	Under visible light, the fouled membranes permeability could be recovered by 99%.
[54] MXene nanosheets and N-doped Bi_2_O_2_CO_3_ nanoparticles (N-BOC)	Chemical etching, ultrasonic-aided exfoliation and vacuum filtration	Hydrophilic	Up to ~ 815.3 L m^−2^ h^−1^	SDS/lubricating oil/H_2_O and SDS/vegetable oil/H_2_O emulsions	>99%	In situ fouling removal via photocatalysis mechanism. The excellent photocatalytic capability of N-BOC was enhanced by the 2D/2D heterojunction structure between MXene and N-BOC.
[63] Ti_3_C_2_T_X_ MXene nanosheets membranes intercalated with sodium alginate	Pre-crosslinking and drop coating	Hydrophilic (WCA = 36°) + Underwater superoleophobic (UOCA ~ 151°)	>42.98 L m^−2^ h^−1^ bar^−1^	Surfactants-stable oil-in-water emulsions n-hexane, octane, toluene	97.22–99.62% -	Extremely low affinity for oil droplets. Significantly lower rejection to anionic dye compared to cationic dye.

**Table 2:** Summary of the main findings for the most recent developments in graphene-based oil–water separation membranes.

Membrane Materials	Synthesis/Fabrication Methods	Wettability	Permeate Flux	Type of Oils Separated	Separation Efficiency/Rejection Rate	Characteristics, Strengths, and Shortcomings
(ii) Graphene						
[89] Graphene and hydrophobic polydimethylsiloxane (PDMS)	Coating on an inexpensive paper tissue	Superhydrophobic (WCA = 153.26°) + Superoleophilic	4421 L m^−2^ h^−1^	Oil/water Mixtures and emulsions Heptane, gasoline, engine oil, soybean oil	99.99% and up to 99.85%	Resistance to acid/alkali, impact, and friction resistance have been greatly improved.
[90] Cell membrane-inspired graphene nanomesh modified with chitosan	Vacuum-aided self-assembly method and synthesized via etching of nanopores on graphene oxide	Hydrophilic + Superoleophobic (UOCA up to 159.8°)	3989 L m^−2^ h^−1^ bar^−1^	Surfactant-stabilized oil-in-water emulsions Silicone oil, sunflower oil, octane, pump oil	98.7%	Membrane is modified with the hydrophilic polymer chitosan to provide a hydration layer that prevents foulants from contacting.
[91] Graphene oxide with phytic acid (PA) and perfluorocarboxylic acids	Sequentially assembled	Hydrophilic + Superoleophobic (UOCA ~ 165°)	~620 L m^−2^ h^−1^ bar^−1^	n-Hexane, hexadecane, vacuum pump oil, corn oil	>98%	An important variable impacting the anti-fouling performance is the perfluoroalkyl chain length because it can adjust the surface hydration structure. Potential toxicity of perfluorocarboxylic acids.
[93] TiO_2_@crumpled graphene oxide core–shell spheres onto electrospun poly (arylene ether nitrile) to obtain fibrous composite membrane	Simple spraying technique	Superhydrophilic + Underwater superoleophobic (UOCA ~ 152–162°)	4830–5160 L m^−2^ h^−1^ (SFE) 3062–3514 L m^−2^ h^−1^ (SSE)	Oil-in-water surfactant-free (SFE) and surfactant-stabilized (SSE) emulsions 1,3,5-trimethyl benzene, isooctane, n-hexane, n-heptane, petroleum ether	>99%	Molecular structure of poly (arylene ether nitrile) was rich in ether bond, benzene ring, and cyano-groups, which gave the membrane a high degree of temperature and corrosion resistance. Structure stability could be efficiently ensured by chemical crosslinking mediated by polydopamine and interactions with TA via hydrogen bonds.
[94] Surfactant-modified graphene oxide (GO-CTAB) on metal meshes	Simple one-step electrodeposition technique	Hydrophilic/ underwater- superoleophobic + Superhydrophobic/oleophilic (switchable)	1800 L m^−2^ h^−1^ (Oil-in-water emulsion) 850 L m^−2^ h^−1^ (Water-in-oil emulsion)	Petroleum ether, n-hexane, n-hexadecane, diesel oil, soybean oil, dichloromethane	~99% for hydrophilic membrane and superhydrophobic membrane (seawater/dichloromethane mixture)	Due to the reduced conjugation and negative electron cloud scattering, GO demonstrated exceptional corrosion resistance.

**Table 3:** Summary of the main findings for the most recent developments in MOF-based oil–water separation membranes.

Membrane Materials	Synthesis/Fabrication Methods	Wettability	Permeate Flux	Type of Oils Separated	Separation Efficiency/Rejection Rate	Characteristics, Strengths, and Shortcomings
(iii) Metal–Organic Framework (MOF)						
[119] MOF-303-coated stainless steel mesh	Simple hydrothermal method to form well-ordered MOF-303 crystals on layered double-hydroxide-modified mesh surface	Superhydrophilic (WCA = 5°) + Superoleophobic (UOCA ~ 151–166°)	12,308–13,300 L m^−2^ h^−1^ (Oil/water mixtures) >2037 L m^−2^ h^−1^ (Emulsions)	Crude oil, cyclohexane, petroleum ether, diesel	>99.35% (Oil/water mixtures) Up to 99.74% (Emulsions)	Remarkable anti-oil-fouling and self-cleaning capabilities. Adsorbed water acted as a cushion to keep oils off the membrane surface.
[114] NH_2_-MIL-88B-coated quartz fibrous membrane	One-step solvothermal method	Superhydrophilic (WCA = 0°) + Superoleophobic (UOCA = 161.3°)	Above 350 L m^−2^ h^−1^ (oil-in-water emulsions)	n-hexane, dichloroethane, petroleum ether, toluene	Up to 99.4%	Good anti-fouling self-cleaning ability. The foulant on the membrane surface can be degraded by Fenton-like catalytic NM88B.
[125] 2D Cu triphenylene catecholate MOF with unique 2D hierarchical structures grown on copper mesh	One-step electrochemical deposition	Superhydrophilic (WCA = 0°) + Underwater superoleophobic (UOCA = 163.1°)	146.3–329.6 k L m^−2^ h^−1^	Cyclohexane, n-hexane, n-pentane, crude oil, petroleum ether, mineral oil, xylene	Less than 24.6 mg L^−1^ of oil residue	Membrane exhibits good recyclability and stability in corrosive environments. As more oil accumulated on the membrane’s surface and impeded the passage of water, the permeability slightly decreased.
[17] Hierarchical micro-/nanostructures prepared by interpenetrating CAU-10-H crystals grown on the mesh	Solvothermal synthesis	Under-liquid dual superlyophobic UW superoleophobic (~147–155°) + UO superhydrophobic (~145–154°) (Switchable)	1.85 × 10^5^ L m^−2^ h^−1^	Immiscible oil/water mixtures and emulsions n-hexane, cyclohexane, methylbenzene, petroleum ether, dichloromethan, chloroform, tetrachloromethane	>99.92%	In diverse hostile environments with alkaline, acidic, and high concentration salt solutions, membrane exhibits exceptional heat and corrosion resistance.
[133] Copper cores and aspartic acid as a ligand + Stearic acid	Electrochemical process	Superhydrophobic textile fabric WCA of 158° ± 1.3, and a water sliding angle of 2° ± 0.2	15,400–15,700 L m^−2^ h^−1^	Oil–water mixtures and emulsions Silicone oil, petroleum ether, n-hexane	95–99.4%	Bio-MOF is a renewable material. Fabric retains its super hydrophobicity after 55 cycles of abrasion, and it also does so after immersion in water solutions with pH range of 5 to 9.

**Table 4:** Summary of the main findings for the most recent developments in COF-based oil–water separation membranes.

Membrane Materials	Synthesis/Fabrication Methods	Wettability	Permeate Flux	Type of Oils Separated	Separation Efficiency/Rejection Rate	Characteristics, Strengths, and Shortcomings
(iv) Covalent Organic Framework (COF)						
[155] Schiff base COFs on electrospun polyacrylonitrile (PAN) nanofibers + Alkyl (Lauryl)	Dip coating	Superhydrophobic (WCA of ~167°) + Oleophilic	Up to ~350 L m^−2^ h^−1^	Suspension of water droplets in oil Paraffin oil, soybean oil, vacuum pump oil, octane	>95%	Stable separation ability after 10 cycles of filtration. Limited information on the fouling resistance of the membrane.
[158] 2D COFs on stainless steel net substrates	Condensation reaction of fluorine and/or isopropyl functional groups and perfluorodialdehyde with triamines	Superhydrophobic (WCA = 150.1°)	~2.84 × 10^5^ L m^−2^ h^−1^	Oil/water mixing systems Petroleum ether, CH_2_Cl_2_, CHCl_3_, *n*-heptane, kerosene, toluene, cyclohexane	>99.5%	Excellent resistance to water, acid, and base, and self-cleaning properties.
[156] Aromatic quinoline-linked COFs	Transforming the dynamic imine linkages into quinoline-linked aromatic rings via aza-Diels–Alder process	Superhydrophobic (WCA up to ~152°)	1.10 × 10^4^–2.20 × 10^4^ L m^−2^ h^−1^	Emulsions Toluene, cyclohexane, chlorobenzene, chloroform	Toluene 89.3%, cyclohexane 86.4%, chlorobenzene 95.3%, chloroform 99.6%	COFs maintained good crystallinity after exposure to acid, base, oxidizing agent, reducing agent, and boiling water.
[159] Imine-linked COFs via condensation of 1,3,5-tris(4-aminophenyl) benzene (TAB) with pyridine-based aldehyde linkers	Microwave-aided synthesis	Superhydrophobic (WCA = 155 ± 2°) + Oleophilic	-	Oil/water separation Pump oil, engine oil, vegetable oil	Removal capacity: Vegetable oil 530 ± 90 wt%, pump oil 518 ± 99 wt%, engine oil 550 ± 80 wt%	Retention of chemical functionalities after immersion in boiling water. Regeneration ability by oleophilic solvents.
[160] Carboxylated COF (COF-COOH) integrated with polydopamine (PDA) assembled on PVDF microfiltration membrane	One-step dip coating method	Superhydrophilic + Underwater superoleophobic (160–165°)	Up to 1843.48 L m^−2^ h^−1^ bar^−1^	Emulsions Diesel, kerosene, soybean oil, n-hexane, petroleum ether	>98%	Good anti-fouling due to the strong electrostatic repulsion induced by carboxyl groups and the robust hydration layer formed by hierarchical nanostructures.

## Data Availability

Not applicable.

## References

[1] Manikandan S., Subbaiya R., Saravanan M., Ponraj M., Selvam M., Pugazhendhi A. (2022). A critical review of advanced nanotechnology and hybrid membrane based water recycling, reuse, and wastewater treatment processes. Chemosphere.

[2] Samuel O., Othman M.H.D., Kamaludin R., Sinsamphanh O., Abdullah H., Puteh M.H., Kurniawan T.A., Li T., Ismail A.F., Rahman M.A. (2022). Oilfield-produced water treatment using conventional and membrane-based technologies for beneficial reuse: A critical review. J. Environ. Manag..

[3] Ismail N.H., Salleh W.N.W., Ismail A.F., Hasbullah H., Yusof N., Aziz F., Jaafar J. (2020). Hydrophilic polymer-based membrane for oily wastewater treatment: A review. Sep. Purif. Technol..

[4] Yang Y., Ali N., Bilal M., Khan A., Ali F., Mao P., Ni L., Gao X., Hong K., Rasool K. (2021). Robust membranes with tunable functionalities for sustainable oil/water separation. J. Mol. Liq..

[5] Baig U., Faizan M., Dastageer M.A. (2021). Polyimide based super-wettable membranes/materials for high performance oil/water mixture and emulsion separation: A review. Adv. Colloid Interface Sci..

[6] Lee H.-J., Choi W.S. (2020). 2D and 3D Bulk Materials for Environmental Remediation: Air Filtration and Oil/Water Separation. Materials.

[7] Yu H., Wu M., Duan G., Gong X. (2022). One-step fabrication of eco-friendly superhydrophobic fabrics for high-efficiency oil/water separation and oil spill cleanup. Nanoscale.

[8] Shalaby M.S., Sołowski G., Abbas W. (2021). Recent Aspects in Membrane Separation for Oil/Water Emulsion. Adv. Mater. Interfaces.

[9] Padaki M., Surya Murali R., Abdullah M.S., Misdan N., Moslehyani A., Kassim M.A., Hilal N., Ismail A.F. (2015). Membrane technology enhancement in oil–water separation. A review. Desalination.

[10] Zhu Y., Wang D., Jiang L., Jin J. (2014). Recent progress in developing advanced membranes for emulsified oil/water separation. NPG Asia Mater..

[11] Cheryan M., Rajagopalan N. (1998). Membrane processing of oily streams. Wastewater treatment and waste reduction. J. Membr. Sci..

[12] Zhang N., Yang X., Wang Y., Qi Y., Zhang Y., Luo J., Cui P., Jiang W. (2022). A review on oil/water emulsion separation membrane material. J. Environ. Chem. Eng..

[13] Tanudjaja H.J., Hejase C.A., Tarabara V.V., Fane A.G., Chew J.W. (2019). Membrane-based separation for oily wastewater: A practical perspective. Water Res..

[14] Junaidi N.F.D., Othman N.H., Fuzil N.S., Mat Shayuti M.S., Alias N.H., Shahruddin M.Z., Marpani F., Lau W.J., Ismail A.F., Aba N.D. (2021). Recent development of graphene oxide-based membranes for oil–water separation: A review. Sep. Purif. Technol..

[15] Diogo Januário E.F., de Camargo Lima Beluci N., Vidovix T.B., Vieira M.F., Bergamasco R., Salcedo Vieira A.M. (2020). Functionalization of membrane surface by layer-by-layer self-assembly method for dyes removal. Process Saf. Environ. Prot..

[16] Chang X., Wang Z., Quan S., Xu Y., Jiang Z., Shao L. (2014). Exploring the synergetic effects of graphene oxide (GO) and polyvinylpyrrodione (PVP) on poly(vinylylidenefluoride) (PVDF) ultrafiltration membrane performance. Appl. Surf. Sci..

[17] Nian P., Wang X., Li S., Wang Z., Wei Y. (2023). Switchable CAU-10-H mesh membrane for on-demand separation of immiscible oil/water mixtures and emulsions. J. Environ. Chem. Eng..

[18] Rasouli S., Rezaei N., Hamedi H., Zendehboudi S., Duan X. (2021). Superhydrophobic and superoleophilic membranes for oil-water separation application: A comprehensive review. Mater. Des..

[19] Ma Q., Cheng H., Fane A.G., Wang R., Zhang H. (2016). Recent Development of Advanced Materials with Special Wettability for Selective Oil/Water Separation. Small.

[20] Shang Y., Si Y., Raza A., Yang L., Mao X., Ding B., Yu J. (2012). An in situ polymerization approach for the synthesis of superhydrophobic and superoleophilic nanofibrous membranes for oil–water separation. Nanoscale.

[21] Wahid F., Zhao X.-J., Duan Y.-X., Zhao X.-Q., Jia S.-R., Zhong C. (2021). Designing of bacterial cellulose-based superhydrophilic/underwater superoleophobic membrane for oil/water separation. Carbohydr. Polym..

[22] Abuhasel K., Kchaou M., Alquraish M., Munusamy Y., Jeng Y.T. (2021). Oily Wastewater Treatment: Overview of Conventional and Modern Methods, Challenges, and Future Opportunities. Water.

[23] Ahmad N.A., Goh P.S., Abdul Karim Z., Ismail A.F. (2018). Thin Film Composite Membrane for Oily Waste Water Treatment: Recent Advances and Challenges. Membranes.

[24] Ali S., Rehman S.A.U., Luan H.-Y., Farid M.U., Huang H. (2019). Challenges and opportunities in functional carbon nanotubes for membrane-based water treatment and desalination. Sci. Total Environ..

[25] Park H.B., Kamcev J., Robeson L.M., Elimelech M., Freeman B.D. (2017). Maximizing the right stuff: The trade-off between membrane permeability and selectivity. Science.

[26] Liu Y., Coppens M.-O., Jiang Z. (2021). Mixed-dimensional membranes: Chemistry and structure–property relationships. Chem. Soc. Rev..

[27] Ihsanullah I., Bilal M. (2022). Recent advances in the development of MXene-based membranes for oil/water separation: A critical review. Appl. Mater. Today.

[28] Kumar C., Das S., Jit S., Jit S., Das S. (2020). 7—Device physics and device integration of two-dimensional heterostructures. 2D Nanoscale Heterostructured Materials.

[29] Shanmugam V., Mensah R.A., Babu K., Gawusu S., Chanda A., Tu Y., Neisiany R.E., Försth M., Sas G., Das O. (2022). A Review of the Synthesis, Properties, and Applications of 2D Materials. Part. Part. Syst. Charact..

[30] Senapati S., Maiti P., Jit S., Das S. (2020). 9—Emerging bio-applications of two-dimensional nanoheterostructure materials. 2D Nanoscale Heterostructured Materials.

[31] Novoselov K.S., Mishchenko A., Carvalho A., Castro Neto A.H. (2016). 2D materials and van der Waals heterostructures. Science.

[32] Cheng Y., Pu Y., Zhao D. (2020). Two-Dimensional Membranes: New Paradigms for High-Performance Separation Membranes. Chem.—Asian J..

[33] Liu G., Jin W., Xu N. (2016). Two-Dimensional-Material Membranes: A New Family of High-Performance Separation Membranes. Angew. Chem. Int. Ed..

[34] Naguib M., Barsoum M.W., Gogotsi Y. (2021). Ten Years of Progress in the Synthesis and Development of MXenes. Adv. Mater..

[35] Dhamodharan D., Dhinakaran V., Byun H.-S. (2022). MXenes: An emerging 2D material. Carbon.

[36] Ihsanullah I. (2020). Potential of MXenes in water desalination: Current status and perspectives. Nano-Micro Lett..

[37] Jiang Q., Lei Y., Liang H., Xi K., Xia C., Alshareef H.N. (2020). Review of MXene electrochemical microsupercapacitors. Energy Storage Mater..

[38] Deysher G., Shuck C.E., Hantanasirisakul K., Frey N.C., Foucher A.C., Maleski K., Sarycheva A., Shenoy V.B., Stach E.A., Anasori B. (2019). Synthesis of Mo4VAlC4 MAX phase and two-dimensional Mo4VC4 MXene with five atomic layers of transition metals. ACS Nano.

[39] Sun Y., Li Y. (2021). Potential environmental applications of MXenes: A critical review. Chemosphere.

[40] Dixit F., Zimmermann K., Dutta R., Prakash N.J., Barbeau B., Mohseni M., Kandasubramanian B. (2022). Application of MXenes for water treatment and energy-efficient desalination: A review. J. Hazard. Mater..

[41] Asif M.B., Iftekhar S., Maqbool T., Paramanik B.K., Tabraiz S., Sillanpää M., Zhang Z. (2021). Two-dimensional nanoporous and lamellar membranes for water purification: Reality or a myth?. Chem. Eng. J..

[42] Kumar J.A., Prakash P., Krithiga T., Amarnath D.J., Premkumar J., Rajamohan N., Vasseghian Y., Saravanan P., Rajasimman M. (2022). Methods of synthesis, characteristics, and environmental applications of MXene: A comprehensive review. Chemosphere.

[43] Maleski K., Alhabeb M. (2019). Top-Down MXene Synthesis (Selective Etching). 2D Metal Carbides and Nitrides (MXenes) Structure, Properties and Applications.

[44] Jiang L., Zhou D., Yang J., Zhou S., Wang H., Yuan X., Liang J., Li X., Chen Y., Li H. (2022). 2D Single-and Few-Layered MXene: Synthesis, applications and perspectives. J. Mater. Chem. A.

[45] Iqbal A., Hong J., Ko T.Y., Koo C.M. (2021). Improving oxidation stability of 2D MXenes: Synthesis, storage media, and conditions. Nano Converg..

[46] Verger L., Xu C., Natu V., Cheng H.-M., Ren W., Barsoum M.W. (2019). Overview of the synthesis of MXenes and other ultrathin 2D transition metal carbides and nitrides. Curr. Opin. Solid State Mater. Sci..

[47] Tao Q., Dahlqvist M., Lu J., Kota S., Meshkian R., Halim J., Palisaitis J., Hultman L., Barsoum M.W., Persson P.O. (2017). Two-dimensional Mo1. 33C MXene with divacancy ordering prepared from parent 3D laminate with in-plane chemical ordering. Nat. Commun..

[48] Zhan X., Si C., Zhou J., Sun Z. (2020). MXene and MXene-based composites: Synthesis, properties and environment-related applications. Nanoscale Horiz..

[49] Xiao X., Yu H., Jin H., Wu M., Fang Y., Sun J., Hu Z., Li T., Wu J., Huang L. (2017). Salt-templated synthesis of 2D metallic MoN and other nitrides. ACS Nano.

[50] Jia J., Xiong T., Zhao L., Wang F., Liu H., Hu R., Zhou J., Zhou W., Chen S. (2017). Ultrathin N-doped Mo2C nanosheets with exposed active sites as efficient electrocatalyst for hydrogen evolution reactions. ACS Nano.

[51] Zhang F., Zhang Z., Wang H., Chan C.H., Chan N.Y., Chen X.X., Dai J.-Y. (2017). Plasma-enhanced pulsed-laser deposition of single-crystalline M o 2 C ultrathin superconducting films. Phys. Rev. Mater..

[52] Zhang Z., Zhang F., Wang H., Chan C.H., Lu W., Dai J.-y. (2017). Substrate orientation-induced epitaxial growth of face centered cubic Mo 2 C superconductive thin film. J. Mater. Chem. C.

[53] Li Z.-K., Liu Y., Li L., Wei Y., Caro J., Wang H. (2019). Ultra-thin titanium carbide (MXene) sheet membranes for high-efficient oil/water emulsions separation. J. Membr. Sci..

[54] Lin Q., Zeng G., Yan G., Luo J., Cheng X., Zhao Z., Li H. (2022). Self-cleaning photocatalytic MXene composite membrane for synergistically enhanced water treatment: Oil/water separation and dyes removal. Chem. Eng. J..

[55] Khatami M., Iravani S. (2021). MXenes and MXene-based materials for the removal of water pollutants: Challenges and opportunities. Comments Inorg. Chem..

[56] Huang Z., Shen L., Lin H., Li B., Chen C., Xu Y., Li R., Zhang M., Zhao D. (2022). Fabrication of fibrous MXene nanoribbons (MNRs) membrane with efficient performance for oil-water separation. J. Membr. Sci..

[57] Hu J., Zhan Y., Zhang G., Feng Q., Yang W., Chiao Y.-H., Zhang S., Sun A. (2021). Durable and super-hydrophilic/underwater super-oleophobic two-dimensional MXene composite lamellar membrane with photocatalytic self-cleaning property for efficient oil/water separation in harsh environments. J. Membr. Sci..

[58] Kong N., Shen L., Zeng Q., Chen C., Teng J., Xu Y., Zhao L., Lin H. (2023). Transversal nanochannel-enabled MXene laminated membranes for superior oil-water separation: A fluid mosaic cytomembrane inspired approach. J. Membr. Sci..

[59] Foller T., Wang H., Joshi R. (2022). Rise of 2D materials-based membranes for desalination. Desalination.

[60] Lin Q., Zeng G., Pu S., Yan G., Luo J., Wan Y., Zhao Z. (2022). A dual regulation strategy for MXene-based composite membrane to achieve photocatalytic self-cleaning properties and multi-functional applications. Chem. Eng. J..

[61] Sun A., Zhan Y., Feng Q., Yang W., Dong H., Liu Y., Chen X., Chen Y. (2022). Assembly of MXene/ZnO heterojunction onto electrospun poly(arylene ether nitrile) fibrous membrane for favorable oil/water separation with high permeability and synergetic antifouling performance. J. Membr. Sci..

[62] Lin Q., Liu Y., Yang Z., He Z., Wang H., Zhang L., Belle Marie Yap Ang M., Zeng G. (2022). Construction and application of two-dimensional MXene-based membranes for water treatment: A mini-review. Results Eng..

[63] Hou K., Zhou H., Zhu K., Xie C., Liu S., He Y., Zhu X., Wu M., Huang T. (2023). Superwetting Ti3C2TX MXene membranes intercalated with sodium alginate for oil/water separation. Carbohydr. Polym. Technol. Appl..

[64] Liu J., Cui L., Losic D. (2013). Graphene and graphene oxide as new nanocarriers for drug delivery applications. Acta Biomater..

[65] Wang Y., Li Z., Wang J., Li J., Lin Y. (2011). Graphene and graphene oxide: Biofunctionalization and applications in biotechnology. Trends Biotechnol..

[66] Yu W., Sisi L., Haiyan Y., Jie L. (2020). Progress in the functional modification of graphene/graphene oxide: A review. RSC Adv..

[67] Tiwari S.K., Sahoo S., Wang N., Huczko A. (2020). Graphene research and their outputs: Status and prospect. J. Sci. Adv. Mater. Devices.

[68] Sanchez V.C., Jachak A., Hurt R.H., Kane A.B. (2012). Biological Interactions of Graphene-Family Nanomaterials: An Interdisciplinary Review. Chem. Res. Toxicol..

[69] Seabra A.B., Paula A.J., de Lima R., Alves O.L., Durán N. (2014). Nanotoxicity of Graphene and Graphene Oxide. Chem. Res. Toxicol..

[70] Pan X., Ji J., Zhang N., Xing M. (2020). Research progress of graphene-based nanomaterials for the environmental remediation. Chin. Chem. Lett..

[71] Papageorgiou D.G., Kinloch I.A., Young R.J. (2015). Graphene/elastomer nanocomposites. Carbon.

[72] Priyadarsini S., Mohanty S., Mukherjee S., Basu S., Mishra M. (2018). Graphene and graphene oxide as nanomaterials for medicine and biology application. J. Nanostruct. Chem..

[73] Geim A.K., Novoselov K.S. (2007). The rise of graphene. Nat. Mater..

[74] Singh V., Joung D., Zhai L., Das S., Khondaker S.I., Seal S. (2011). Graphene based materials: Past, present and future. Prog. Mater. Sci..

[75] Balandin A.A., Ghosh S., Bao W., Calizo I., Teweldebrhan D., Miao F., Lau C.N. (2008). Superior Thermal Conductivity of Single-Layer Graphene. Nano Lett..

[76] Lee C., Wei X., Kysar J.W., Hone J. (2008). Measurement of the elastic properties and intrinsic strength of monolayer graphene. Science.

[77] McAllister M.J., Li J.-L., Adamson D.H., Schniepp H.C., Abdala A.A., Liu J., Herrera-Alonso M., Milius D.L., Car R., Prud’homme R.K. (2007). Single Sheet Functionalized Graphene by Oxidation and Thermal Expansion of Graphite. Chem. Mater..

[78] Upadhyay R.K., Soin N., Roy S.S. (2014). Role of graphene/metal oxide composites as photocatalysts, adsorbents and disinfectants in water treatment: A review. RSC Adv..

[79] Novoselov K.S., Geim A.K., Morozov S.V., Jiang D.-e., Zhang Y., Dubonos S.V., Grigorieva I.V., Firsov A.A. (2004). Electric field effect in atomically thin carbon films. Science.

[80] Hernandez Y., Nicolosi V., Lotya M., Blighe F.M., Sun Z., De S., McGovern I.T., Holland B., Byrne M., Gun’Ko Y.K. (2008). High-yield production of graphene by liquid-phase exfoliation of graphite. Nat. Nanotechnol..

[81] Whitener K.E., Sheehan P.E. (2014). Graphene synthesis. Diam. Relat. Mater..

[82] Charrier A., Coati A., Argunova T., Thibaudau F., Garreau Y., Pinchaux R., Forbeaux I., Debever J.-M., Sauvage-Simkin M., Themlin J.-M. (2002). Solid-state decomposition of silicon carbide for growing ultra-thin heteroepitaxial graphite films. J. Appl. Phys..

[83] Li X., Cai W., An J., Kim S., Nah J., Yang D., Piner R., Velamakanni A., Jung I., Tutuc E. (2009). Large-area synthesis of high-quality and uniform graphene films on copper foils. Science.

[84] Bae S., Kim H., Lee Y., Xu X., Park J.-S., Zheng Y., Balakrishnan J., Lei T., Ri Kim H., Song Y.I. (2010). Roll-to-roll production of 30-inch graphene films for transparent electrodes. Nat. Nanotechnol..

[85] Luo X., He Z., Gong H., He L. (2022). Recent advances in oil-water separation materials with special wettability modified by graphene and its derivatives: A review. Chem. Eng. Process.—Process Intensif..

[86] Guo F., Zhang C., Wang Q., Hu W., Cao J., Yao J., Jiang L., Wu Z. (2019). Modification of poly (vinylidene fluoride) membranes with aluminum oxide nanowires and graphene oxide nanosheets for oil–water separation. J. Appl. Polym. Sci..

[87] Yuliwati E., Ismail A.F. (2011). Effect of additives concentration on the surface properties and performance of PVDF ultrafiltration membranes for refinery produced wastewater treatment. Desalination.

[88] Nishigochi S., Ishigami T., Maruyama T., Hao Y., Ohmukai Y., Iwasaki Y., Matsuyama H. (2014). Improvement of antifouling properties of polyvinylidene fluoride hollow fiber membranes by simple dip coating of phosphorylcholine copolymer via hydrophobic interactions. Ind. Eng. Chem. Res..

[89] Sun J., Jia H., Bi H., Que M., Chen L., Zhang Q., Xiong Y., Xie X., Sun Y. (2022). Laser-assisted synthesis of graphene-based paper for both oil/water mixtures and emulsions separation. Process Saf. Environ. Prot..

[90] Liu Y., Coppens M.-O. (2022). Cell Membrane-Inspired Graphene Nanomesh Membrane for Fast Separation of Oil-in-Water Emulsions. Adv. Funct. Mater..

[91] Yang C., Long M., Ding C., Zhang R., Zhang S., Yuan J., Zhi K., Yin Z., Zheng Y., Liu Y. (2022). Antifouling graphene oxide membranes for oil-water separation via hydrophobic chain engineering. Nat. Commun..

[92] Galli G., Martinelli E. (2017). Amphiphilic polymer platforms: Surface engineering of films for marine antibiofouling. Macromol. Rapid Commun..

[93] Chen X., Zhan Y., Sun A., Feng Q., Yang W., Dong H., Chen Y., Zhang Y. (2022). Anchoring the TiO2@crumpled graphene oxide core–shell sphere onto electrospun polymer fibrous membrane for the fast separation of multi-component pollutant-oil–water emulsion. Sep. Purif. Technol..

[94] Yang J., Sun J., Wang Z., Wang L. (2022). Surfactant-modified graphene oxide complex-coating functionalized material with robust switchable oil/water wettability for high-performance on-demand emulsion separation. Surf. Coat. Technol..

[95] Ma L., Wang J., Li J., Pang Y., He J., Peng L., Li Y., Li K., Qu M. (2021). Intelligent composite foam with reversible tunable superwettability for efficient and sustainable oil/water separation and high-concentration organic wastewater purification. Process Saf. Environ. Prot..

[96] Yan T., Zhang T., Zhao G., Zhang C., Li C., Jiao F. (2019). Magnetic textile with pH-responsive wettability for controllable oil/water separation. Colloids Surf. A Physicochem. Eng. Asp..

[97] Awwad M., Bilal M., Sajid M., Nawaz M.S., Ihsanullah I. (2023). MOF-based membranes for oil/water separation: Status, challenges, and prospects. J. Environ. Chem. Eng..

[98] Li J., Wang H., Yuan X., Zhang J., Chew J.W. (2020). Metal-organic framework membranes for wastewater treatment and water regeneration. Coord. Chem. Rev..

[99] Gangu K.K., Maddila S., Mukkamala S.B., Jonnalagadda S.B. (2016). A review on contemporary Metal–Organic Framework materials. Inorg. Chim. Acta.

[100] Kuppler R.J., Timmons D.J., Fang Q.-R., Li J.-R., Makal T.A., Young M.D., Yuan D., Zhao D., Zhuang W., Zhou H.-C. (2009). Potential applications of metal-organic frameworks. Coord. Chem. Rev..

[101] Furukawa H., Cordova K.E., O’Keeffe M., Yaghi O.M. (2013). The Chemistry and Applications of Metal-Organic Frameworks. Science.

[102] Safaei M., Foroughi M.M., Ebrahimpoor N., Jahani S., Omidi A., Khatami M. (2019). A review on metal-organic frameworks: Synthesis and applications. TrAC Trends Anal. Chem..

[103] Li X., Yang X., Xue H., Pang H., Xu Q. (2020). Metal–organic frameworks as a platform for clean energy applications. EnergyChem.

[104] Xuan W., Zhu C., Liu Y., Cui Y. (2012). Mesoporous metal–organic framework materials. Chem. Soc. Rev..

[105] Qiu S., Xue M., Zhu G. (2014). Metal–organic framework membranes: From synthesis to separation application. Chem. Soc. Rev..

[106] Zhu M., Liu Y., Chen M., Sadrzadeh M., Xu Z., Gan D., Huang Z., Ma L., Yang B., Zhou Y. (2021). Robust superhydrophilic and underwater superoleophobic membrane optimized by Cu doping modified metal-organic frameworks for oil-water separation and water purification. J. Membr. Sci..

[107] Lu W., Duan C., Zhang Y., Gao K., Dai L., Shen M., Wang W., Wang J., Ni Y. (2021). Cellulose-based electrospun nanofiber membrane with core-sheath structure and robust photocatalytic activity for simultaneous and efficient oil emulsions separation, dye degradation and Cr (VI) reduction. Carbohydr. Polym..

[108] Geng Q., Dong S., Li Y., Wu H., Yang X., Ning X., Yuan D. (2022). High-Performance photoinduced antimicrobial membrane toward efficient PM2.5-0.3 capture and Oil-Water separation. Sep. Purif. Technol..

[109] Lu W., Duan C., Liu C., Zhang Y., Meng X., Dai L., Wang W., Yu H., Ni Y. (2020). A self-cleaning and photocatalytic cellulose-fiber- supported “Ag@AgCl@MOF- cloth’’ membrane for complex wastewater remediation. Carbohydr. Polym..

[110] Gao J., Wei W., Yin Y., Liu M., Zheng C., Zhang Y., Deng P. (2020). Continuous ultrathin UiO-66-NH2 coatings on a polymeric substrate synthesized by a layer-by-layer method: A kind of promising membrane for oil–water separation. Nanoscale.

[111] Gholami F., Zinadini S., Zinatizadeh A.A. (2020). Preparation of high performance CuBTC/PES ultrafiltration membrane for oily wastewater separation; A good strategy for advanced separation. J. Environ. Chem. Eng..

[112] Zhang R., Cao J., Liu Y.-N., Guan J., He M., Jiang Z. (2020). Metal–organic framework-intercalated graphene oxide membranes for highly efficient oil/water separation. Ind. Eng. Chem. Res..

[113] Zhu X., Yu Z., Liu Y., Li X., Long R., Wang P., Wang J. (2021). NH2-MIL-125@ PAA composite membrane for separation of oil/water emulsions and dyes. Colloids Surf. A Physicochem. Eng. Asp..

[114] Xie A., Wu Y., Liu Y., Xue C., Ding G., Cheng G., Cui J., Pan J. (2022). Robust antifouling NH2-MIL-88B coated quartz fibrous membrane for efficient gravity-driven oil-water emulsion separation. J. Membr. Sci..

[115] Xiang X., Chen D., Li N., Xu Q., Li H., He J., Lu J. (2022). Mil-53 (Fe)-loaded polyacrylonitrile membrane with superamphiphilicity and double hydrophobicity for effective emulsion separation and photocatalytic dye degradation. Sep. Purif. Technol..

[116] Song P., Lu Q. (2020). Porous clusters of metal-organic framework coated stainless steel mesh for highly efficient oil/water separation. Sep. Purif. Technol..

[117] Baker R.W. (2002). Future Directions of Membrane Gas Separation Technology. Ind. Eng. Chem. Res..

[118] Deng Y., Wu Y., Chen G., Zheng X., Dai M., Peng C. (2021). Metal-organic framework membranes: Recent development in the synthesis strategies and their application in oil-water separation. Chem. Eng. J..

[119] Yin X., He Y., He T., Li H., Wu J., Zhou L., Li S., Li C. (2023). A durable MOF-303-coated stainless steel mesh with robust anti-oil-fouling performance for multifunctional oil/water separation. Colloids Surf. A Physicochem. Eng. Asp..

[120] Ezazi M., Shrestha B., Kim S.-I., Jeong B., Gorney J., Hutchison K., Lee D.H., Kwon G. (2020). Selective Wettability Membrane for Continuous Oil−Water Separation and In Situ Visible Light-Driven Photocatalytic Purification of Water. Glob. Chall..

[121] Shrestha B., Ezazi M., Kwon G. (2021). Engineered Nanoparticles with Decoupled Photocatalysis and Wettability for Membrane-Based Desalination and Separation of Oil-Saline Water Mixtures. Nanomaterials.

[122] Xie A., Cui J., Yang J., Chen Y., Lang J., Li C., Yan Y., Dai J. (2020). Photo-Fenton self-cleaning PVDF/NH2-MIL-88B(Fe) membranes towards highly-efficient oil/water emulsion separation. J. Membr. Sci..

[123] Yang B., Ding L., Yao H., Chen Y., Shi J. (2020). A Metal-Organic Framework (MOF) Fenton Nanoagent-Enabled Nanocatalytic Cancer Therapy in Synergy with Autophagy Inhibition. Adv. Mater..

[124] Guerin T.F. (2000). Long-term performance of a land treatment facility for the bioremediation of non-volatile oily wastes. Resour. Conserv. Recycl..

[125] He X.-T., Li B.-Y., Liu J.-X., Tao W.-Q., Li Z. (2022). Facile fabrication of 2D MOF-Based membrane with hierarchical structures for ultrafast Oil-Water separation. Sep. Purif. Technol..

[126] Jin L., Wang Y., Xue T., Xie J., Xu Y., Yao Y., Li X. (2019). Smart amphiphilic random copolymer-coated sponge with pH-switchable wettability for on-demand oil/water separation. Langmuir.

[127] Fu Y., Jin B., Zhang Q., Zhan X., Chen F. (2017). pH-induced switchable superwettability of efficient antibacterial fabrics for durable selective oil/water separation. ACS Appl. Mater. Interfaces.

[128] Zong C., Hu M., Azhar U., Chen X., Zhang Y., Zhang S., Lu C. (2019). Smart copolymer-functionalized flexible surfaces with photoswitchable wettability: From superhydrophobicity with “rose petal” effect to superhydrophilicity. ACS Appl. Mater. Interfaces.

[129] Zhu H., Yang S., Chen D., Li N., Xu Q., Li H., He J., Lu J. (2016). A robust absorbent material based on light-responsive superhydrophobic melamine sponge for oil recovery. Adv. Mater. Interfaces.

[130] Fan S., Li Z., Fan C., Chen J., Huang H., Chen G., Liu S., Zhou H., Liu R., Feng Z. (2022). Fast-thermoresponsive carboxylated carbon nanotube/chitosan aerogels with switchable wettability for oil/water separation. J. Hazard. Mater..

[131] Kung C.H., Zahiri B., Sow P.K., Mérida W. (2018). On-demand oil-water separation via low-voltage wettability switching of core-shell structures on copper substrates. Appl. Surf. Sci..

[132] Nadar S.S., Vaidya L.B., Maurya S.S., Rathod V.K. (2019). Polysaccharide based metal organic frameworks (polysaccharide–MOF): A review. Coord. Chem. Rev..

[133] Mohamed M., Abd-El-Nabey B. (2022). Fabrication of a biological metal–organic framework based superhydrophobic textile fabric for efficient oil/water separation. Sci. Rep..

[134] Waller P.J., Gándara F., Yaghi O.M. (2015). Chemistry of Covalent Organic Frameworks. Acc. Chem. Res..

[135] Feng X., Ding X., Jiang D. (2012). Covalent organic frameworks. Chem. Soc. Rev..

[136] Ding S.-Y., Wang W. (2013). Covalent organic frameworks (COFs): From design to applications. Chem. Soc. Rev..

[137] Guan X., Chen F., Fang Q., Qiu S. (2020). Design and applications of three dimensional covalent organic frameworks. Chem. Soc. Rev..

[138] Wang Z., Zhang S., Chen Y., Zhang Z., Ma S. (2020). Covalent organic frameworks for separation applications. Chem. Soc. Rev..

[139] Zhang C., Wu B.-H., Ma M.-Q., Wang Z., Xu Z.-K. (2019). Ultrathin metal/covalent–organic framework membranes towards ultimate separation. Chem. Soc. Rev..

[140] Khayum M.A., Kandambeth S., Mitra S., Nair S.B., Das A., Nagane S.S., Mukherjee R., Banerjee R. (2016). Chemically Delaminated Free-Standing Ultrathin Covalent Organic Nanosheets. Angew. Chem. Int. Ed..

[141] Kandambeth S., Biswal B.P., Chaudhari H.D., Rout K.C., Kunjattu H.S., Mitra S., Karak S., Das A., Mukherjee R., Kharul U.K. (2017). Selective Molecular Sieving in Self-Standing Porous Covalent-Organic-Framework Membranes. Adv. Mater..

[142] Yang H., Wu H., Yao Z., Shi B., Xu Z., Cheng X., Pan F., Liu G., Jiang Z., Cao X. (2018). Functionally graded membranes from nanoporous covalent organic frameworks for highly selective water permeation. J. Mater. Chem. A.

[143] Yuan S., Li X., Zhu J., Zhang G., Van Puyvelde P., Van der Bruggen B. (2019). Covalent organic frameworks for membrane separation. Chem. Soc. Rev..

[144] Lei R., Zha Z., Hao Z., Wang J., Wang Z., Zhao S. (2022). Ultrathin and high-performance covalent organic frameworks composite membranes generated by oligomer triggered interfacial polymerization. J. Membr. Sci..

[145] Dey K., Pal M., Rout K.C., Kunjattu H.S., Das A., Mukherjee R., Kharul U.K., Banerjee R. (2017). Selective molecular separation by interfacially crystallized covalent organic framework thin films. J. Am. Chem. Soc..

[146] Wang X., Shi B., Yang H., Guan J., Liang X., Fan C., You X., Wang Y., Zhang Z., Wu H. (2022). Assembling covalent organic framework membranes with superior ion exchange capacity. Nat. Commun..

[147] Zhang K., He Z., Gupta K.M., Jiang J. (2017). Computational design of 2D functional covalent–organic framework membranes for water desalination. Environ. Sci. Water Res. Technol..

[148] Shen J., Yuan J., Shi B., You X., Ding R., Zhang T., Zhang Y., Deng Y., Guan J., Long M. (2021). Homointerface covalent organic framework membranes for efficient desalination. J. Mater. Chem. A.

[149] Huang N., Zhai L., Xu H., Jiang D. (2017). Stable Covalent Organic Frameworks for Exceptional Mercury Removal from Aqueous Solutions. J. Am. Chem. Soc..

[150] Ding S.-Y., Dong M., Wang Y.-W., Chen Y.-T., Wang H.-Z., Su C.-Y., Wang W. (2016). Thioether-Based Fluorescent Covalent Organic Framework for Selective Detection and Facile Removal of Mercury(II). J. Am. Chem. Soc..

[151] Ning G.-H., Chen Z., Gao Q., Tang W., Chen Z., Liu C., Tian B., Li X., Loh K.P. (2017). Salicylideneanilines-Based Covalent Organic Frameworks as Chemoselective Molecular Sieves. J. Am. Chem. Soc..

[152] Shinde D.B., Sheng G., Li X., Ostwal M., Emwas A.-H., Huang K.-W., Lai Z. (2018). Crystalline 2D Covalent Organic Framework Membranes for High-Flux Organic Solvent Nanofiltration. J. Am. Chem. Soc..

[153] Qahtan T.F., Gondal M.A., Dastageer M.A., Kwon G., Ezazi M., Al-Kuban M.Z. (2020). Thermally sensitized membranes for crude oil–water remediation under visible light. ACS Appl. Mater. Interfaces.

[154] Shrestha B., Ezazi M., Rad S.V., Kwon G. (2021). Predicting kinetics of water-rich permeate flux through photocatalytic mesh under visible light illumination. Sci. Rep..

[155] Chen A., Guo H., Zhou J., Li Y., He X., Chen L., Zhang Y. (2022). Polyacrylonitrile nanofibers coated with covalent organic frameworks for oil/water separation. ACS Appl. Nano Mater..

[156] Wang Y., Xie J., Ren Z., Guan Z.-H. (2022). Postsynthetically modified hydrophobic covalent organic frameworks for enhanced oil/water and CH_4_/C_2_H_2_ separation. Chem. Eng. J..

[157] Wu X., Hong Y.-L., Xu B., Nishiyama Y., Jiang W., Zhu J., Zhang G., Kitagawa S., Horike S. (2020). Perfluoroalkyl-Functionalized Covalent Organic Frameworks with Superhydrophobicity for Anhydrous Proton Conduction. J. Am. Chem. Soc..

[158] Liu Y., Li W., Yuan C., Jia L., Liu Y., Huang A., Cui Y. (2022). Two-Dimensional Fluorinated Covalent Organic Frameworks with Tunable Hydrophobicity for Ultrafast Oil–Water Separation. Angew. Chem. Int. Ed..

[159] Das G., Skorjanc T., Prakasam T., Garai B., Abubakar S., Zalch C.S., Gándara F., Pasricha R., Sharma S.K., Varghese S. (2022). Hydrophobicity Tuning in Isostructural Urchin-Shaped Covalent Organic Framework Nanoparticles by Pore Surface Engineering for Oil–Water Separation. ACS Appl. Nano Mater..

[160] Liang Q., Jiang B., Yang N., Zhang L., Sun Y., Zhang L. (2022). Superhydrophilic Modification of Polyvinylidene Fluoride Membrane via a Highly Compatible Covalent Organic Framework–COOH/Dopamine-Integrated Hierarchical Assembly Strategy for Oil–Water Separation. ACS Appl. Mater. Interfaces.

[161] Zhang S., Bilal M., Adeel M., Barceló D., Iqbal H.M. (2021). MXene-based designer nanomaterials and their exploitation to mitigate hazardous pollutants from environmental matrices. Chemosphere.

[162] Li J., Li Y., Lu Y., Wang Y., Guo Y., Shi W. (2023). Preparation of 2D Materials and Their Application in Oil—Water Separation. Biomimetics.

